# Yield gap analyses to estimate attainable bovine milk yields and evaluate options to increase production in Ethiopia and India

**DOI:** 10.1016/j.agsy.2017.04.007

**Published:** 2017-07

**Authors:** Dianne Mayberry, Andrew Ash, Di Prestwidge, Cécile M. Godde, Ben Henderson, Alan Duncan, Michael Blummel, Y. Ramana Reddy, Mario Herrero

**Affiliations:** a CSIRO Agriculture and Food, 306 Carmody Rd, St Lucia, Qld 4067, Australia; b International Livestock Research Institute, PO Box 5689, Addis Ababa, Ethiopia; c Sri Venkateswara Veterinary University, Tirupati, India

**Keywords:** Buffalo, Cattle, Dairy, Grasslands, Food security

## Abstract

Livestock provides an important source of income and nourishment for around one billion rural households worldwide. Demand for livestock food products is increasing, especially in developing countries, and there are opportunities to increase production to meet local demand and increase farm incomes. Estimating the scale of livestock yield gaps and better understanding factors limiting current production will help to define the technological and investment needs in each livestock sector. The aim of this paper is to quantify livestock yield gaps and evaluate opportunities to increase dairy production in Sub-Saharan Africa and South Asia, using case studies from Ethiopia and India. We combined three different methods in our approach. Benchmarking and a frontier analysis were used to estimate attainable milk yields based on survey data. Household modelling was then used to simulate the effects of various interventions on dairy production and income. We tested interventions based on improved livestock nutrition and genetics in the extensive lowland grazing zone and highland mixed crop-livestock zones of Ethiopia, and the intensive irrigated and rainfed zones of India. Our analyses indicate that there are considerable yield gaps for dairy production in both countries, and opportunities to increase production using the interventions tested. In some cases, combined interventions could increase production past currently attainable livestock yields.

## Highlights

• Three methods were used to quantify livestock yield gaps and evaluate interventions.
• Large datasets were used to estimate attainable milk production.
• Household modelling highlights ways to increase production.
• The biggest increases in production occur when multiple interventions are combined.
• Interventions need to consider production goals and reasons farmers keep livestock.

## 1 Introduction

The demand for livestock food products, including dairy, in developing countries is projected to double in the next forty years as we see an increase in the human population, incomes and urbanisation (Herrero and Thornton, 2013). At the same time, the amount of land and water available for agriculture is decreasing and many grazing lands are becoming increasingly degraded (McDermott et al., 2010). Smallholder farmers need to increase production of dairy products to satisfy household demands for nutrition and income, but also to stay competitive in the face of growing competition from large-scale producers. This increase in production needs to be achieved through more efficient and sustainable farming systems (Anderson et al., 2016).

Most of the world's poor tropical livestock producers are in Sub-Saharan Africa and South Asia. Here, dairy is produced in mixed crop-livestock systems ranging from low-input, extensive grazing to more specialised, intensive enterprises (Herrero et al., 2010, McDermott et al., 2010). Household incomes and nutritional security could be raised through increased livestock production in these areas. Estimating the extent to which production can be increased and better understanding factors currently limiting production will help to define the technological and investment needs in each sector. In this paper we explore the use of yield gap analysis to investigate the potential to increase household yields and income from dairy production in Sub-Saharan Africa and South Asia, using examples from Ethiopia and India.

### 1.1 Dairy production in Ethiopia and India

#### 1.1.1 Ethiopia

Ethiopia is ranked tenth in the world for population of ruminant livestock with 55 million cattle and 56 million sheep and goats (Central Statistics Agency, 2014). Almost all cattle are local breeds, with crossbred and exotic breeds such as Holstein-Friesian and Jerseys comprising only 1.3% of the national cattle herd. Crossbred cattle are mostly used in commercial milk production, and are concentrated in the highland mixed crop-livestock region. In the agro-pastoral and pastoral lowland regions, most milk is produced by multi-purpose indigenous livestock that also provide draught and beef. Total national milk production is 2.9 billion litres per year but average milk production per cow is low at 1.4 l/head/day over a 6 month lactation. Much of the milk produced is for home consumption or for sale at local markets, with only 5% of milk produced sold through commercial markets (USAID, 2013).

#### 1.1.2 India

India is the largest milk producer in the world, producing 130 billion kg milk/year (Ministry of Agriculture, 2014b, Rao et al., 2014). Milk and other dairy products account for around two thirds of the value of the Indian livestock sector and support the livelihoods of nearly half of India's 147 million rural households. Dairy production is concentrated in the irrigated cropping zone, but large amounts of milk are also produced in rainfed areas. The majority of the cattle population consists of indigenous breeds, which have low milk yields of around 2 kg/head/day (Ministry of Agriculture, 2014a, Ministry of Agriculture, 2014b). Most milk comes from buffalo, which comprise one third of the bovine population and produce around 5 kg milk/head/day (Kumar and Parappurathu, 2014). Cattle diets are based almost entirely on crop residues and by-products, with contributions from grazing of natural pastures (Rao et al., 2014, Singh et al., 2008). Sub-optimal milk production is driven by low daily milk yields, short lactations (6–8 months) and long calving intervals (18–24 months) (Duncan et al., 2013).

### 1.2 Livestock yield gaps

Yield gap analyses are used to estimate the extent to which agricultural production can be increased for a particular farm or region, and to identify factors constraining production. Yield gaps are regularly reported for cropping systems at the field scale, but are less commonly applied to livestock production systems. However, application to livestock production systems is significant, since they are an important component of smallholder farming systems. Recent examples of livestock yield gap analyses include Cortez-Arriola et al. (2014), van der Linden et al. (2015) and Henderson et al. (2016).

A yield gap is typically defined as the difference between actual and potential or water-limited yields for an agricultural product. Actual yield is the average yield for a specific location, and potential yield is the maximum achievable yield under best management practices, including use of irrigation where it is available (van Ittersum et al., 2013). Water-limited yields are used to indicate the maximum achievable yields for rainfed systems where crops are not irrigated. In developing countries such as Ethiopia and India, access to farm inputs is often limited and yield gap analyses based on potential or water-limited yields from optimal use of resources and high inputs can create unrealistic expectations of how much production can be increased in practice. In this context it may be more useful to consider relative yield gaps. Relative yield gaps are the difference between actual and attainable yields, where attainable yields are the maximum yields achievable given locally available resources and technologies (Tittonell and Giller, 2013, van der Linden et al., 2015).

In the literature there is a variety of methods for calculating potential and attainable yields, and they can be broadly characterised as modelled potential yields or potential yields based on statistical analyses of survey reported yields. A frequent criticism of modelled potential yields is that they are often based on optimal farming conditions (e.g. crops are sown at optimal sowing time with effective pest, weed and disease control), and ignore practical farm-level constraints (Anderson et al., 2016, Dzanku et al., 2015, Neumann et al., 2010, van Ittersum et al., 2013). However, this method can give insights into factors constraining production and how current yields might be improved. In addition, a well parameterised model can be used to predict potential yields within specific constraints (e.g. access to livestock feed). Conversely, while potential yields based on statistical analysis of reported data (e.g. Licker et al. (2010)) may give a more realistic expectation of what is locally achievable, there is rarely enough detail in the data to provide insights into how farm yields can be increased. Many statistical analyses also focus only on potential increases in farm yield, without considering the impacts on household income or food security. This is important because farmers have no incentive to increase yields unless there is an overall benefit to the household. Such analyses may also ignore socio-economic constraints such as availability of labour, prices of inputs and access to markets.

In this study, we used a combination of methods to quantify relative yield gaps for bovine dairy in Ethiopia and India, and evaluate strategies to increase production and household income from livestock within the constraints of existing resources. This information will contribute to making informed investment decisions and target technologies in the livestock sectors of developing countries (Herrero et al., 2015).

## 2 Methods

For the purpose of this study, we defined livestock yield gaps as the difference between actual and attainable yields, given locally available resources and technologies. We used benchmarking and frontier analysis of reported yields to define actual and attainable milk yields in Ethiopia and India, and thereby calculate the size of the relative yield gaps. Results from this analysis were used to inform the household modelling. Household modelling was used to evaluate strategies to increase dairy production within the constraints of the current production systems, and indicate likely economic outcomes. We specifically included profit from livestock production in our analysis because farmers are unlikely to adopt new technologies or changes to their farming practices unless there is a net benefit to their household.

### 2.1 Data

Data about dairy production were obtained from a number of databases, a summary of which is provided in Table 5 in the Supplementary section. As a brief overview, the OPEC database compiled by Duncan et al. (2013), IMPACT Lite (CGIAR research program on Climate Change Agriculture and Food Security, Rufino et al. (2013)) and Living Standards Measurement Study (LSMS, The World Bank) datasets provided information on both Ethiopian and Indian dairy production. The Village Dynamics in South Asia (VDSA, ICRISAT) database provided additional data about Indian production systems. Government census information was also available for both countries (Central Statistics Agency, 2014, Ministry of Agriculture, 2014a, Ministry of Agriculture, 2014b, Ministry of Agriculture, 2015). These databases provided information on the number of livestock per household, milk production, reproduction, mortality, feeding, and production of crops. Additional data about livestock management and pricing were gained from the literature (Gupta et al., 2014, Kumar and Parappurathu, 2014, Kumar and Kumar, 2013, Meena et al., 2015, Rao et al., 2014, Singh et al., 2008, Tegegne et al., 2013).

### 2.2 Benchmarking and frontier analyses

Two different but related methods were used to estimate relative yield gaps based on farm survey data. With each approach farms within sample populations are compared with their most productive peers. Accordingly, these methods attempt to measure potential yield improvements from better use of existing practices rather than from adopting new practices that can radically improve productivity. The two methods require different types of data, so different datasets were used for each analysis. This meant that we were not able to compare the same regions (Ethiopia) or states (India) between analyses.

In the first method, a simple benchmarking approach was used to estimate the potential for improving milk yields in a particular agricultural zone. This involved comparing average milk yields of the top 10% most productive farms with the average yield for the whole of the sample population. This was done for a number of different locations in Ethiopia (LSMS database, 2003) and India (VSDA database, 2013). Data was not disaggregated based on cattle breeds.

The second approach was to estimate yield gaps based on the construction of production frontiers for mixed crop-livestock smallholder farmers in Ethiopia (LSMS and IMPACT lite databases) and India (IMPACT lite and VSDA databases). Detailed methods for frontier analysis are available in Henderson et al. (2016). Briefly, a frontier is constructed for each site based on the most efficient farms in each site. The frontiers consider all farm inputs and outputs and describe the maximum level of output that could be achieved by the farms in each site. A single technical efficiency score is then estimated for each farm based on the gap between the observed and maximum attainable output possible for each farm given their existing basket of inputs. The scores are radial measures of technical efficiency, based on the simultaneous expansion of all farm outputs. The frontiers were constructed using a parametric estimation method known as Stochastic Frontier Analysis (SFA) estimation procedure (Aigner et al., 1977, Meeusen and van den Broeck, 1977). The SFA frontier describes the maximum possible level of production given the amount of all production inputs used in the sample population, taking into account both statistical noise and technical inefficiency; the latter causing farms to lie below the frontier. We used a multi-output distance function approach to handle the multiple outputs present in our farming systems. We used maximum-likelihood methods to estimate the stochastic Cobb-Douglas distance functions with the usual distributional assumptions, namely, the random error variables are independently and identically distributed normal random variables, and the inefficiency variables are nonnegative, independently and identically distributed half-normal random variables. We also tested the hypothesis that inefficiency effects are absent from the model: given the distributional assumptions above and maximum-likelihood estimation method used, a simple z-test (defined as the mean value of the parameter representing inefficiency effects in the model divided by its standard error) was used for this purpose (Coelli et al., 2005). The SFA methodology has sound theoretical underpinnings in production economics and has been used in other published yield gap assessments (Henderson et al., 2016, Neumann et al., 2010) as have other similar production frontier methods (Baldos and Hertel, 2012, Nin-Pratt et al., 2011). Estimation was carried out using the FRONTIER econometric package developed by Coelli and Henningsen (2015) for implementation in R software.

### 2.3 Household modelling case studies

#### 2.3.1 Model setup and baseline scenarios

Interventions to address livestock yield gaps were evaluated using a smallholder household simulation model run over a 20 year period. The integrated analysis tool (IAT, version 1.3.7) (Lisson et al., 2010) is a spreadsheet model that integrates crop production, forages, livestock production, herd dynamics, household economics and labour supply. It has previously been used to model both intensive (cut and carry) and extensive (grazing) livestock production systems in East Asia (China, Indonesia, Vietnam), South and West Asia (India, Pakistan), and Africa (Burkina Faso, Cameroon, Niger, Senegal, Zimbabwe) (e.g. Komarek et al., 2012, Parsons et al., 2011, Rigolot et al., 2017, Shafiullah, 2012).

The IAT was parameterised to create baseline scenarios for dairy production across Ethiopia and India. Baseline scenarios were developed to reflect characteristics of a typical production enterprise in each region, based on review of databases and literature described above. Details of each baseline scenario are described in Table 1. In Ethiopia, dairy production was simulated for three agro-ecological zones as defined by the Ethiopia Livestock Master Plan (Shapiro et al., 2015). These were: Lowland Grazing (LG) in pastoral zones (< 900 mm rainfall) based largely on grazing of natural pastures, highland Mixed crop-livestock Rainfall Deficient (MRD) zone where rainfall is 900–1400 mm and households are rural with some crop land and access to grazing, and the highland Mixed crop-livestock Rainfall Sufficient (MRS) zone where rainfall is > 1400 mm. For this study the household type in the MRS was based on peri-urban and urban livestock producers who do not have access to land. In India, dairy production was simulated in the irrigated and rainfed zones. The irrigated zone is characterised by intensive crop and livestock production, and most milk is produced from buffalo in a cut and carry system. The rainfed zone is less intensive, and most cattle are indigenous breeds.

**Table 1:** Characteristics of baseline dairy production households in different agro-ecological regions of Ethiopia and India, and details of simulated interventions. Feed weights are fresh weights. LG: lowland grazing pastoral zone; MRD: mixed crop-livestock rainfall deficient zone; MRS: mixed crop-livestock rainfall sufficient zone.

Scenario & interventions	Description	Livestock breed	Number breeders
**Ethiopia-LG-dairy**	Lowland grazing zone. Extensive grazing system with no cropping land. Cattle grazing natural pasture. Cows supplemented with 0.4 kg noug cake/head/day. Baseline herd mortality 5%. Male offspring sold at 12 months/180 kg.	Zebu	15–30
Improved pasture	N content of pasture increased (to simulate reseeding with legumes). No change to supplementation.	Zebu	15–20
			
**Ethiopia-MRD-dairy**	Highland mixed farming in rainfall deficient zone. Grazing of natural grasses from 0.8 ha land. Cereal straw grown from 1 ha cropping land fed as required. Cows supplemented with 0.4 kg noug cake/head/day and purchased legume hay as required. Baseline herd mortality 10%. Male offspring sold at 1 month.	Zebu	3–5
Concentrate	Noug cake increased to 0.8 kg/head/day. Additional supplement of 0.8 kg wheat bran/head/day.	Zebu	3–5
Improved forage	0.5 ha natural pasture replaced with *Lablab purpureus*. No change to supplementation from baseline.	Zebu	3–5
Improved genetics	Zebu cattle replaced by crossbred cattle. Fed cereal straw and purchased legume hay as required, noug cake at 0.8 kg/head/day.	Crossbred cattle	4–8
Improved genetics + improved forage	Zebu cattle replaced by crossbred cattle. 0.7 ha natural pasture replaced with *Lablab purpureus*.	Crossbred cattle	4–6
Improved genetics + concentrate	Zebu cattle replaced by crossbred cattle. Cows supplemented with 1.5 kg/head/day noug cake + 1.5 kg/head/day wheat bran.	Crossbred cattle	3–6
			
**Ethiopia-MRS-dairy**	Highland mixed farming in rainfall sufficient zone. Urban dairy production with no cropping or grazing land. Cows fed cereal straw, legume hay, 0.4 kg noug cake/head/day. Baseline herd mortality 10%. Male offspring sold at 1 month.	Zebu	3–4
Improved forage	N content of cereal straw increased with urea. No change to supplementation.	Zebu	3–4
Concentrate	Noug cake increased to 0.8 kg/head/day noug cake. Additional supplement of 0.8 kg wheat bran/head/day.	Zebu	3–4
Improved genetics	Zebu cattle replaced with crossbred cattle. Cows fed cereal straw, legume hay, 0.8 kg noug cake/head/day.	Crossbred cattle	4–5
Improved genetics + improved forage	Zebu cattle replaced with crossbred cattle. N content of cereal straw increased with urea. Noug cake increased to 1.0 kg/head/day.	Crossbred cattle	4–5
Improved genetics + concentrate	Zebu cattle replaced with crossbred cattle. Noug cake increased to 1.6 kg/head/day. Additional supplement of 1.6 kg wheat bran/head/day.	Crossbred cattle	4–5
			
**India-rainfed-dairy**	Rainfed zone. 1 ha of cropping land used to grow maize, sorghum and wheat. Cattle grazed native grass, supplemented with 4 kg crop residues/head/day. Baseline mortality 5%. Male offspring sold at 1 month.	Local cattle	1–3
Improved genetics (buffalo)	Local cattle replaced with buffalo. Buffalo graze native grass, supplemented with 5 kg crop residues/head/day.	Buffalo	1–3
Improved genetics (crossbred cattle)	Local cattle replaced with crossbred cattle. Cattle graze native grass, supplemented with 6 kg crop residues/head/day.	Crossbred cattle	1–3
Low concentrate	Cattle supplemented with 1 kg wheat bran/head/day.	Local cattle	1–3
Improved genetics (buffalo) + low concentrate	Local cattle replaced with buffalo. Buffalo graze native grass, supplemented with 5 kg crop residues and 1 kg wheat bran/head/day.	Buffalo	1–3
Improved genetics (crossbred cattle) + low concentrate	Local cattle replaced with crossbred cattle. Cattle graze native grass, supplemented with 6 kg crop residues and 1 kg wheat bran/head/day.	Crossbred cattle	1–3
Improved genetics (buffalo) + high concentrate	Local cattle replaced with buffalo. Buffalo graze native grass, supplemented with 5 kg crop residues and 3 kg wheat bran/head/day.	Buffalo	1–3
Improved genetics (crossbred cattle) + high concentrate	Local cattle replaced with crossbred cattle. Cattle graze native grass, supplemented with 6 kg crop residues and 3 kg wheat bran/head/day.	Crossbred cattle	1–3
			
**India-irrigated-dairy**	Irrigated zone. 0.5 ha cropping land used to grow rice and wheat. Cows fed cereal straw and native grass. Supplemented with 1.5 kg rice bran/head/day. Baseline mortality 5%. Male offspring sold at 1 month.	Buffalo	1–3
Improved forage	Quality of cereal straw increased by 1 MJ metabolisable energy/kg DM. Price of straw increased from 5 to 5.8 INR/kg.	Buffalo	1–3
Green feed	Buffalo supplemented with 10 kg good quality grass/head/day.	Buffalo	1–3
Green feed + bran	Buffalo supplement with 8 kg good quality grass/head/day. Bran increased to 3 kg/head/day.	Buffalo	1–3
Increased bran	Bran increased to 5 kg/head/day.	Buffalo	1–3

The livestock simulation model within the IAT predicts the liveweight gain and reproduction cycles for ruminants under specified local feeding and husbandry practices. Herd size within the IAT is based on a minimum and maximum number of females of breeding age (breeders) set by the user, with management rules to sell livestock based on age and weight of different livestock classes. Livestock production within the IAT is based on energy and protein supply in the diet using the Feeding Standards for Australian Livestock (Freer et al., 2007). Default livestock breeds are available within the model, but can be edited to reflect characteristics of local breeds. Livestock breeds and mature weights included in our simulations were Zebu cattle (350 kg), crossbred cattle (420 kg), and indigenous cattle (350 kg) in Ethiopia, and crossbred cattle (500 kg) and buffalo (450 kg) in India.

For the modelling we assumed that livestock are fed through grazing of pastures and stall-feeding of forages, crop resides, crop by-products and purchased supplements. Where sufficient climate, soil and management information was available, crop and forage production were simulated in detailed, stand-alone models such the Agricultural Production System Simulator (APSIM; (Keating et al., 2003)) or GRASP (McKeon et al., 1990) and imported into the IAT. Grazed pastures for extensive production systems were simulated using GRASP, driven by daily climate variables simulated for current climate using the MarkSim weather generator (Jones and Thornton, 2000). However, there was insufficient information available to model crop production, so yields and monthly availability of crops (grain and residues) and improved forages were estimated based on information available in the databases described above. Crop residues were stockpiled at harvest and available until the supply was exhausted, when additional feeds were purchased if necessary.

Annual profit (income–expenses) is calculated by the IAT model, and was only considered for the livestock component of the farming system. Income was gained from the sale of milk and livestock (culled breeders, offspring), and values attributed to these are specified in Table 6 (Supplementary section). The value of livestock for provision of manure, draught power and transport was not considered as there was insufficient data available in the literature to parameterise this section of the model. Costs included purchasing feed for livestock if feed production on-farm was insufficient, health care and mating costs. The cost of producing livestock feed was not included because the majority of feeds are either by-products of crop production (straw, stovers, brans) or cut/grazed from communal lands.

Labour was not explicitly considered in our analyses because there was insufficient information available in the literature to parameterise the model. It was assumed that enough family labour was available to sustain livestock production.

#### 2.3.2 Interventions to increase dairy production

Interventions evaluated in this study included (1) improving livestock nutrition, and (2) replacing indigenous livestock with improved breeds. These interventions were chosen because they represented the most effective way of increasing livestock productivity i.e. increasing growth rate, increasing livestock numbers and preventing losses. Details of each scenario are described in Table 1, with a broad overview in the text below.

We investigated several options for improving livestock nutrition. For extensive dairy production we explored increasing both the quality and quantity of feed resources. To improve the quality of communal grazing lands we simulated reseeding of natural pasture with a perennial, herbaceous legume (e.g. Stylosanthes) by increasing the N content of the available forage by 0.5%. The seasonal decline in nitrogen content of pasture was also reduced to simulate the higher protein content maintained in grass-legume pastures when grasses mature and senesce. It is recognised that augmentation of native pastures with a legume will not be relevant to all systems, but it can be a relatively low cost way of improving the feedbase. The costs of establishing an improved pasture are usually borne by the farmer, but because pasture areas are communal grazing lands it was assumed that the government would provide the investment for pasture improvement and no cost to the producer was included in our modelling. In more intensive production scenarios, nutrition was primarily improved by increasing the amount and quality of supplements offered to livestock. Supplements included high quality forages (grass and legumes), urea-treated crop residues, and concentrate-type feeds or crop by-products (e.g. noug cake and cereal brans). In the Indian dairy scenario we also modelled increasing the nutritive value of crop residues by growing improved cultivars with higher metabolisable energy content (Anandan et al., 2013, Bidinger and Blümmel, 2007, Blummel and Rao, 2006, Reddy et al., 2003). In cases with insufficient farm availability of feed, additional feeds were purchased. Quality and costs of supplements are described in Table 7 (Supplementary section). Feed was not offered ad libitum as our experience is that this is not common in smallholder farming systems.

Replacing local livestock breeds with crossbred cattle or buffalo was investigated for both countries. Improved breeds have higher production potential and sale value, but also higher liveweight, feed requirements and production costs (Duncan et al., 2013, Kumar and Kumar, 2013, Leroy et al., 2016, Ministry of Agriculture, 2014b). Buffalo had higher milk fat content compared to both local and crossbred cattle, which requires additional energy to produce but also generally attracts a higher sale price (Squicciarini and Vandeplas, 2011).

## 3 Results

### 3.1 Benchmarking and frontier analyses

Milk yields (Table 2) and technical efficiency (Table 3) were higher in India compared to Ethiopia, although the absolute size of the yield gap expressed as kg milk/head/lactation was also larger in India. Within both countries, technical efficiency was higher in the rainfed (India) and MRD (Ethiopia) regions compared to the higher-producing MRS (Ethiopia) and irrigated (India) areas.

**Table 2:** Dairy yield and yield gaps estimated using benchmarking analysis. MRS: mixed crop-livestock rainfall sufficient zone; MRD: mixed crop-livestock rainfall deficient zone; LG: lowland grazing pastoral zone; SNNP: Southern Nations, Nationalities and People's Region.

Country	Agricultural zone	State/region in data set	Milk yields (kg/head/lactation)		Yield gap	
			Top 10% households	All households	(kg/head/lactation)	(% increase)
Ethiopia	LG	Benishangul gumuz, Gambela, Somalie	597	279	318	114
	MRD	Tigray, SNNP	627	213	414	194
	MRS	Dire Dawa, Harari, Oromiya	1000	314	686	219
India	Rainfed	Andhra Pradesh, Jharkhand, Karnataka, Maharastra, Madhya Pradesh, Orissa	2304	639	1666	261
	Irrigated	Bihar	2812	1159	1653	143

**Table 3:** Mean technical efficiency (the average of the individual farm technical efficiency scores) and yield gaps of milk production estimated using stochastic frontier analysis, and the results of a hypothesis test – null hypothesis specifies that inefficiency effects are absent from the model. MRS: mixed crop-livestock rainfall sufficient zone; MRD: mixed crop-livestock rainfall deficient zone; LG: lowland grazing pastoral zone; SNNP: Southern Nations, Nationalities and People's Region.

Country	Agro-climatic zone	Region/state	Mean technical efficiency	Test statistic (z-value)	Yield gap (% increase)
Ethiopia	MRD	SNNP	0.68	2.28^c^	49
	MRS/MRD	Amhara	0.38	3.65^a^	151
	MRS	Oromiya	0.43	3.54^a^	130
India	Rainfed	Andhra Pradesh	0.69	2.27^c^	45
	Irrigated	Haryana	0.60	1.13^d^	55
	Irrigated	Bihar	0.61	2.28^c^	72

a, b, c and d indicate the level of statistical significance: a (< 0.001); b (< 0.01); c (< 0.05); d (< 0.1).

In comparing the two methods, the yield gaps measured as potential percentage improvements were appreciably higher for the simple benchmarking approach (Table 2) than when estimated using the frontier efficiency approach (Table 3).

### 3.2 Household modelling

#### 3.2.1 Ethiopia

Milk production per cow was lowest in the baseline scenario for the lowland grazing region (292 kg/head/lactation, Table 4), but financial returns were positive as a result of revenue from sales of cattle. Improving the quality of grazed pasture by reseeding with legumes increased milk production to 407 kg/head/lactation, but milk yields remained low compared to the highland regions (MRD, MRS). Improved financial returns from legume reseeding were mostly a result of increased cattle production rather than improvements in milk production per head. Higher herd numbers could be sustained, there was a much reduced calving interval, and a nearly 300% increase in cattle sales.

**Table 4:** Modelled interventions to increase dairy production in Ethiopia and India. Scenarios are ranked by annual milk production per farm within each site. Profit is from livestock production only. 1 USD = 22 ETB or 66 INR.

Region × scenario	Herd size (heads)	Calving interval (months)	Milk yield (kg/cow/lactation)	Milk yield (kg/cow/yr)	Milk yield (kg/farm/yr)	Turnoff (heads/yr)^a^	Mortality (%)	Annual profit
**Ethiopia-LG-Dairy**								ETB

Baseline	26.3	26	292	124	1617	4.1	14	8496
Improved pasture	38.4	15	407	273	4724	11.6	8	27,411

**Ethiopia-MRD-Dairy**								ETB

Baseline	4.2	24	306	153	321	0.6	13	− 104
Concentrate	5.1	18	669	446	1419	1.5	9	2488
Improved forage	5.3	22	702	380	1140	0.9	10	3687
Improved genetics	4.9	20	741	326	945	0.6	19	676
Improved genetics + improved forage	6.6	18	1753	958	3927	1.4	8	16,058
Improved genetics + concentrate	7.4	14	1960	1404	6879	2.6	7	33,406

**Ethiopia-MRS-Dairy**								ETB

Baseline	4.3	24	429	208	519	0.8	10	− 1388
Improved forage	5.6	14	474	403	1129	1.5	10	30
Concentrate	5.1	12	627	649	1817	2.1	8	4149
Improved genetics	5.5	20	848	454	1544	1.0	10	947
Improved genetics + improved forage	6.2	16	1810	1157	4488	1.6	7	17,680
Improved genetics + concentrate	6.6	12	1885	1506	6626	2.7	6	31,003

**India-rainfed-Dairy**								INR

Baseline	3.7	20	379	223	638	1.3	8	7776
Improved genetics (buffalo)	3.7	21	650	370	1060	1.2	8	30,911
Improved genetics (crossbred cattle)	3.6	22	864	460	1286	1.1	8	15,948
Low concentrate	3.7	18	703	452	1311	1.5	8	12,592
Improved genetics (buffalo) + low concentrate	3.7	19	1050	646	1883	1.4	7	54,834
Improved genetics (crossbred) + low concentrate	3.7	20	1214	727	2106	1.4	7	22,869
Improved genetics (buffalo) + high concentrate	3.8	18	1796	1189	3514	1.7	5	96,639
Improved genetics (crossbred) + high concentrate	3.8	18	1990	1294	3809	1.6	5	34,543

**India-irrigated-dairy**								INR

Baseline	3.7	20	1025	613	1757	1.3	8	25,637
Improved forage	3.7	19	1311	835	2448	1.5	6	56,122
Green feed	3.9	20	2050	1206	3617	1.5	5	74,774
Green feed + bran	3.8	18	2284	1498	4417	1.6	6	95,477
Increased bran	3.8	18	2636	1702	5000	1.6	6	116,850

a Includes sale of male offspring, female calves not needed as replacement breeders, and culled mature breeders.

In both highland systems, feeding natural pasture and/or crop residues with 0.4 kg noug cake/head/day (baseline) to indigenous Zebu cattle resulted in low milk yields (306 and 429 kg/head/lactation for MRD and MRS, respectively), long calving intervals (24 months) and financial losses (Table 4). The losses were greater in the urban households because all fodders were purchased. Feeding additional concentrate and improved forages to indigenous cows lifted productivity as cow weight and condition improved. This increased reproductive performance through reduced calving interval and lower mortality.

Replacing indigenous zebu with crossbred cattle resulted in a doubling of milk yields, even on baseline diets, and farms shifted from loss making enterprises to ones making a small profit (Table 4). Milk yields from crossbred cattle were further increased to 1753–1960 kg/head/lactation when feeding was improved through provision of improved forages and concentrate feeds. This was accompanied by a decrease in calving interval and mortality rates. The resulting increase in sale of livestock contributed to the increase in profit.

#### 3.2.2 India

Baseline milk production from local cattle in the rainfed zone was only 379 kg/head/lactation (Table 4). Improving livestock nutrition doubled milk yields, whilst also reducing calving intervals, resulting in an increase in annual milk production and profit. Replacing local cattle breeds with buffalo or crossbred cattle also increased milk yields, but the biggest increases in production and income were achieved by replacing local cattle with buffalo or crossbred cattle and providing a high quality concentrate supplement. Replacing local cattle with buffalo provided the biggest increases in income, even though the level of production was not as high as for crossbred cattle.

Baseline milk production in the irrigated zone of India was higher than in the rainfed zone, at 1025 kg milk/head/lactation. Improving the feeding value of crop residues and supplementing she-buffalo with additional green feed or concentrates increased average milk production up to 2636 kg/head/lactation, whilst also decreasing calving intervals and mortality rates (Table 4). Milk production and income increased relative to the quality of the diet (metabolisable energy and N content), with high energy feeds such as rice and wheat bran providing the biggest and most cost-efficient increases.

## 4 Discussion

While there is a large amount of research into agronomic yield gaps, there is limited information available on livestock yield gaps in Sub-Saharan Africa and South Asia. Quantification of livestock yield gaps is a relatively new field, and as yet there is no standard methodology. By combining different yield gap analysis methods we were able to calculate relative yield gaps for dairy production in Ethiopia and India based on attainable yields (benchmarking and frontier analysis), and evaluate possible interventions to increase production (household modelling). Large yield gaps exist for dairy production in both countries, and packages of interventions are required to bridge these gaps rather than single interventions.

### 4.1 Quantification of livestock yield gaps

Statistical analysis of reported data provided a useful complement to the household modelling by quantifying baseline levels of production and locally attainable yields. Differences in the size of yield gaps calculated with the benchmarking (Table 2) and frontier analysis (Table 3) approaches reflect important differences between methods. The simple benchmarking approach is used to estimate the maximum amount of milk that could be produced per head, taking into consideration the use of other farm inputs. Therefore, the yield improvement suggested by the simple approach may be associated with either more intensive use of inputs, such as feed and land, or from a more efficient use of these and other farm resources. By contrast, the frontier approach places much greater restrictions on the potential pathways for farms to improve their performance, by only allowing yield gaps to be reduced by improving the efficiency (or management) with which each farm's observed bundle of inputs are used, and thus excluding the potential gains from intensifying the use of any of these inputs. Consequently, the more restrictive frontier approach tends to generate lower estimates of potential yield gap improvement.

While the yield gaps based on the simple benchmarking approach are higher than those based on frontier analysis for both countries, the differences between the two approaches are much greater for India than for Ethiopia. Notwithstanding the different regional aggregations used in each approach, this result suggests more intensive use of non-livestock inputs can increase yields more dramatically, compared to when inputs are held constant, in the Indian sites compared to the Ethiopian sites. Considering the benchmarking approach as an upper bound on what can be achieved with access to existing practices, there is considerable scope to increase milk yields in both regions. However, without intensification the scope for improvements is understandably much lower, especially in India.

Probably the biggest limitation of these methods for calculating yield gaps is the availability of good quality data. Both types of analyses require large datasets, and our analyses had to be done at a regional level rather than the farm level because limited data was available. If more data was available, these analyses could be conducted at a finer scale, and provide information on the scale of yield gaps for specific areas or farm types.

### 4.2 Evaluation of interventions based on simulation modelling to increase dairy production

Comparison of yield gaps between the benchmarking analysis (Table 2) and household modelling (Table 4) provided insights into how dairy farmers could increase milk production to levels similar to those reached by the top producers in each agricultural zone. For the India rainfed and irrigated scenarios, maximum attainable yields were similar between the benchmarking and household modelling. Maximum modelled yields were 86 and 94% of the benchmarking yields for the rainfed and irrigated zones, respectively. This indicates that improved genetics and nutrition could be enough to close the yield gap for dairy production in India. Additional interventions not tested in our modelling could increase production past what the top producers are currently achieving. For the other scenarios, maximum yields from simulated interventions were either substantially lower (Ethiopia LG, 407 vs 597 kg/cow/lactation) or higher (Ethiopia MRD, 1960 vs 617 kg/cow/lactation and MRS, 1885 vs 1000 kg/cow/lactation) than the maximum attainable yields indicated in the benchmarking exercise. Where yields were lower, additional interventions (e.g. more feed, better disease management and healthcare, improved mating services) may be required to further increase production. In comparison, for the highland dairy systems in Ethiopia, only single interventions such as improved feeding or genetics were required to fill the yield gap indicated by the benchmarking analysis. For these scenarios it is worth highlighting that the baseline milk production in the household modelling was around 100 kg/head/lactation higher than that indicated in the benchmarking, and additional factors not included in our modelling may be limiting production. Simulation of combined nutrition and genetics interventions indicates that it may be possible to increase production past currently attainable yields. However, there may be good reasons why even the top producers have not reached this level of production, and a better understanding of local conditions is required before implementing interventions.

An important outcome of the household modelling activity was to highlight that interventions with the highest yields of milk did not always have the highest profits. For example, in the rainfed dairy scenario in India (Table 4), replacing local cattle with buffalo was more profitable than using crossbred cattle, despite crossbred cattle having higher milk yields. This is because (1) buffalo milk has a higher fat content and value compared to cow's milk, and (2) male cattle have no value because they cannot be legally slaughtered in most Indian states (Kumar and Kumar, 2013, Squicciarini and Vandeplas, 2011). Profit is an important consideration when designing intervention packages. Once household consumption needs have been met, additional milk can be sold and income spent on items and services that provide benefits to the household (e.g. healthcare, education, labour-saving devices, additional crop inputs).

Whilst the input costs of the interventions were captured in the modelling, one of the limitations of our analysis was that we were unable to determine the availability of additional labour requirements for implementing different interventions. This is important because labour supply is often one of the key limitations to intensifying agricultural production (Herrero et al., 2014, Squicciarini and Vandeplas, 2011). Some of the interventions modelled in our study may increase labour demands, limiting the opportunity for family members to participate in off- or non-farm work (Tittonell et al., 2010), which often contributes substantially to household income. Labour shortages can also impact the timing of key activities such as planting of crops and livestock insemination, reducing potential farm yields and income. A shortage of labour can be addressed by hiring labour, but this relies on farmers having access to cash or other methods of payment, and farmers may need to forfeit expenditure on other items (e.g. fertiliser for crops) (Tittonell et al., 2010). Hiring labour would also decrease the profit from livestock production estimated in our modelling (Table 4). Conversely, some interventions tested in our modelling could reduce labour requirements. Ashley et al. (2016) found that growing plots of improved forages near the house decreased labour requirements for raising cattle because less time was spent cutting grass from roadsides and/or herding cattle for grazing. Reduced labour requirements particularly benefited the children and women of the household, who were then able to spend more time at school or on other income-generating activities.

### 4.3 Application of results

Borrowing yield gap analysis approaches from the agronomic research community and applying them to livestock production has considerable utility. However, some caution needs to be exercised because of the multi-purpose character of livestock keeping in developing countries (Swanepoel et al., 2010, Weiler et al., 2014). While we focused on milk production because of the contribution to household food security, smallholder farmers have multiple reasons for keeping livestock. In this context, increasing milk yields is only one of a number of production objectives for smallholder farmers. Farmers need to balance measures to increase productivity with risk mitigation, use of livestock to supply draught power and manure, the role of livestock as capital assets and so on. Proposals for reducing yield gaps need then to be viewed through the lens of the multi-functionality of livestock in developing country contexts.

The increases in individual livestock productivity indicated by the modelling and benchmarking analysis are in line with national policy goals. For example, the Ethiopian Livestock Master Plan has targets for the year 2020 of increasing individual dairy cow milk production from 247 l/year to 1053 l/year (Shapiro et al., 2015). The household modelling in this study suggest that with a combination of improved genetics and better use of forages these individual animal gains are achievable (Table 4). In both India and Ethiopia, the required improvements in individual productivity were most effectively achieved by combining technologies rather than through a singular focus on genetics or nutrition. This need for system improvements rather than individual technologies is consistent with other analyses in tropical livestock systems (Ash et al., 2015).

Whilst the opportunities to lift ruminant productivity through improved nutrition and genetics appear compelling from the analysis in this study, the challenges associated with adoption and implementation should not be under-estimated. These include cost, technical capacity, social and cultural barriers, and attitudes to risk. The interventions discussed in this paper require capital investment in new livestock, and increased spending on livestock feed, mating and healthcare. In many situations there is not the credit available for smallholders to be able to adopt the new technologies – and even if the credit is available, farmers are often risk averse when it comes to taking on loans. There is also the challenge of technical capacity to successfully implement the interventions. This is exacerbated by poor or inappropriately targeted extension efforts. There is also risk associated with climatic variability that might limit the successful implementation of interventions, especially those associated with improving the feedbase. This whole area of credit, risk and technical capacity requires further attention and should be addressed holistically rather than as individual components if livestock productivity and profitability is to be improved in Sub-Saharan Africa and South Asia.

## 5 Conclusion

Different methods for analysing yield gaps can be combined to give estimates of locally attainable yields for livestock products and evaluate possible interventions to increase production and profits. Our analysis showed that there are considerable yield gaps for dairy production in areas of Ethiopia and India. The scale of the yield gaps indicates that there are opportunities to increase production within the constraints of current production systems. It also appears possible to increase production past currently attainable yields. Household modelling showed that milk yields, reproduction, growth rates and survival can be improved through better nutrition and genetics, but the biggest increases will be realised when multiple strategies are combined. This information can be used by governments, development agencies and donors to make informed investment decisions.

## Appendix A Supplementary data

Supplementary material Image 1
