# Supplementary material for: Yield gap analyses to estimate attainable bovine milk yields and evaluate options to increase production in Ethiopia and India
Source: Agric Syst. 2017 Jul;155:43–51. doi: 10.1016/j.agsy.2017.04.007 (PMC5485639; doi:10.1016/j.agsy.2017.04.007)
Supplement: Supplementary file 1 — Supplementary material [file mmc1.docx]

## 8. Supplementary data

Table 5. Overview of datasets used in yield gap analysis. Some datasets contain additional information not described here, which was not used in our project.

| **Dataset** | **Reference or source** | **Year** | **Location** |  | **Types of data** |
| --- | --- | --- | --- | --- | --- |
| Agricultural Sample Survey | Central Statistical Agency, 2014 | 2013-14 | Ethiopia (all regions) |  | Livestock number by species, sex, age, breed, purpose  Milk production (daily yield, length of lactation)  Disease & vaccination rates  Number livestock/hh  Types of feed |
| Agricultural statistics at a glance | Ministry of Agriculture, 2015 | 2014 | India (all states) |  | Crops planted (area, yield) |
| Basic Animal Husbandry and Fisheries Statistics | Ministry of Agriculture, 2014b | 2012-13 | India (all states) |  | Milk production (daily yield), number lactating livestock  Number livestock slaughtered, meat yield/head  Number of livestock by species, breed, sex  Disease rates |
| IMPACT lite | Rufino et al., 2013 | 2012 | Ethiopia (Borana)  India (Bihar, Haryana) |  | Land area  Labour  Farm assets  Livestock numbers  Livestock yields and inputs  Crop yields and inputs |
| Livestock Census | Ministry of Agriculture, 2014a | 2012 | India (all states) |  | Livestock number by species, breed, sex, age, purpose, reproductive & lactation status |
| Living Standards Measurement Study | World Bank  <http://econ.worldbank.org/WBSITE/EXTERNAL/EXTDEC/EXTRESEARCH/EXTLSMS/0,,menuPK:3359053~pagePK:64168427~piPK:64168435~theSitePK:3358997,00.html> | 2011-12 (Ethiopia)  1997-98 (India) | Ethiopia (Afar, Amhara, Benishangul Gumuz, Dire Dawa, Gambella, Harari, Oromiya, Southern Nations Nationalities and Peoples' Region (SNNP), Somalie, Tigray)  India (Bihar, Uttar Pradesh) |  | Land area  Number of livestock  Crop grown (type, area, yield, inputs, value)  Labour |
| OPEC | Duncan et al., 2013 | 2010 | Ethiopia (Amhara, Oromiya)  India (Bihar, Punjab, Andhra Pradesh) |  | Land area  Number of livestock by breed  Milk yield  Feeding practices (time spent grazing, types of feed, amount fed)  Feed purchased and produced by household  Feed value  Problems with livestock health, reproduction and milk production |
| Village Dynamics in South Asia | ICRISAT  <http://vdsa.icrisat.ac.in> | 2009 onwards | Andhra Pradesh, Bihar, Gujarat, Jharkhand, Karnataka, Madhya Pradesh, Maharashtra, Orissa |  | Land area  Crop production  Number of livestock  Types and amount of feed fed  Milk yields and prices |

Table 6. Livestock management costs (excluding feed) and sale prices of livestock products

|  | **Costs** |  |  | **Income** |  |  |
| --- | --- | --- | --- | --- | --- | --- |
| **Country x Breed** | **Healthcare** | **Mating** |  | **Milk** | **Female livestock** | **Male livestock** |
|  | Per head per month | Per year |  | Per kg milk | Per kg liveweight | Per kg liveweight |
| *Ethiopia (ETB)* |  |  |  |  |  |  |
| Zebu cattle-LG | 5 | 0 |  | 8 | 8 | 8 |
| Zebu cattle-MRD | 4 | 10 |  | 8 | 10 | 10 |
| Zebu cattle-MRS | 4 | 50 |  | 8 | 10 | 10 |
| Crossbred cattle | 4 | 100 |  | 8 | 10 | 10 |
|  |  |  |  |  |  |  |
| *India (INR)* |  |  |  |  |  |  |
| Indigenous cattle | 100 | 400 |  | 30 | 50 | 0 |
| Crossbred cattle | 150 | 600 |  | 30 | 60 | 0 |
| Buffalo | 150 | 400 |  | 50 | 60 | 15 |

Table 7. Feed quality parameters used by the IAT model and prices of purchased feed (fresh weight basis)

| **Feed type** | **Dry matter**  **(%)** | **Dry matter digestibility**  **(%)** | **N content**  **(% DM)** | **Cost per kg** |
| --- | --- | --- | --- | --- |
| *Ethiopia* |  |  |  | *ETB* |
| Cereal straws | 90 | 45 | 0.7 | 0.3 |
| Lablab hay | 90 | 60 | 2.0 | - |
| Noug seed cake | 90 | 70 | 5.1 | 1.8 |
| Pulse straw | 90 | 55 | 1.8 | 0.5 |
| Urea-treated stover | 90 | 57 | 3 | 0.6 |
| Wheat bran | 89 | 80 | 2.5 | 1.7 |
|  |  |  |  |  |
| *India* |  |  |  | *INR* |
| Cereal straws-rainfed | 90 | 46 | 0.7 | 4 |
| Cereal straws-irrigated | 90 | 46 | 0.7 | 5 |
| Cereal straws-high energy content | 90 | 53 | 0.7 | 5.8 |
| Good quality grass | 20 | 60 | 1.5 | 2.5 |
| Maize stover | 90 | 52 | 0.6 | 5 |
| Rice bran | 90 | 70 | 2.4 | 16 |
| Sorghum stover | 90 | 50 | 0.6 | 5 |
| Wheat bran | 90 | 69 | 2.8 | 16 |
